# Heterodimerization of Two Pathological Mutants Enhances the Activity of Human Phosphomannomutase2

**DOI:** 10.1371/journal.pone.0139882

**Published:** 2015-10-21

**Authors:** Giuseppina Andreotti, Maria Chiara Monti, Valentina Citro, Maria Vittoria Cubellis

**Affiliations:** 1 Istituto di Chimica Biomolecolare –CNR, Pozzuoli, Italy; 2 Dipartimento di Farmacia, Università degli Studi di Salerno, Fisciano, Italy; 3 Dipartimento di Biologia, Università Federico II, Napoli, Italy; Consejo Superior de Investigaciones Cientificas, SPAIN

## Abstract

The most frequent disorder of glycosylation is due to mutations in the gene encoding phosphomannomutase2 (PMM2-CDG). For this disease, which is autosomal and recessive, there is no cure at present. Most patients are composite heterozygous and carry one allele encoding an inactive mutant, R141H, and one encoding a hypomorphic mutant. Phosphomannomutase2 is a dimer. We reproduced composite heterozygosity *in vitro* by mixing R141H either with the wild type protein or the most common hypomorphic mutant F119L and compared the quaternary structure, the activity and the stability of the heterodimeric enzymes. We demonstrated that the activity of R141H/F119L heterodimers *in vitro*, which reproduces the protein found in patients, has the same activity of wild type/R141H, which reproduces the protein found in healthy carriers. On the other hand the stability of R141H/F119L appears to be reduced both *in vitro* and *in vivo*. These findings suggest that a therapy designed to enhance protein stability such as those based on pharmacological chaperones or modulation of proteostasis could be beneficial for PMM2-CDG patients carrying R141H/F119L genotype as well as for other genotypes where protein stability rather than specific activity is affected by mutations.

## Introduction

The most frequent disorder affecting the transfer of N-linked oligosaccharides to proteins is caused by a deficiency of Phosphomannomutase2 (UniProt: PMM2_HUMAN): accordingly the recommended name for this congenital disorder of glycosylation is PMM2-CDG (MIM#212065), but the same pathology is also known as CDG Ia or Jaeken syndrome.

The major activity of PMM2 *in vivo* consists in the isomerization of mannose 6-phosphate into mannose 1-phosphate which is activated and eventually introduced into glycans. *In vitro* PMM2 isomerizes glucose 6-phosphate too although with a lower specific activity and contrary to what was observed for the paralogous enzyme phosphomannomutase1 [1], PMM2 has little, if any, phosphatase activity. The crystal structure of the human enzyme deposited in the PDB contains only one subunit in the asymmetric unit. The PISA server [2] provides a possible biological assembly and an estimation of the subunit surface area, 7.6%, that becomes inaccessible to solvents when the protein dimerizes. Dimeric structure is needed for PMM2 activity [3]. The precise mechanism of catalysis is not yet known and, since the known substrates are small molecules, the reason why dimeric state is needed is not clear.

PMM2-CDG is transmitted through autosomal recessive inheritance and its clinical spectrum is wide with neurological symptoms and a variable involvement of other organs. More than 85 missense mutations have been described for the gene encoding PMM2 [4] which is located on chromosome 16 (16p13.2). R141H (NM_000303.2: c.422G>A; NP_000294.1: p.Arg141His) is the most frequent mutation and was detected in 8 out of 1000 in a default global population (1000Genome phase 1 genotype data from 1094 worldwide individuals). It is present in about 75% of the alleles of central European patients and is recurrent in certain populations, the frequency of carriers being 1 out of 60 and 1 out of 79 in Danish and Dutch populations respectively [5]. R141 is located in the active site of the enzyme where it is predicted to bind the phosphate group of the substrate, mannose-6-phosphate [6]. Its mutation into histidine, which completely inactivates the enzyme, has never been observed in homozygosity possibly because the total absence of PMM2 is not compatible with life [7]. Carriers are healthy thus indicating that 50% residual activity is sufficient to prevent symptoms.

The second most frequent mutation, F119L (NM_000303.2:c.357C>A; NP_000294.1: p.Phe119Leu) is common in Scandinavia as well as in other Northern European countries and its incidence decreases moving from the North to the South of the continent. The mutation of F119 into leucine weakens the quaternary structure, which is required for activity, and causes a reduction of the enzymatic activity. Both R141H and F119L are described in DBSNP [8], a database that stores polymorphisms as well as pathological mutations, with the codes rs28936415 and rs80338701 respectively.

F119L, as other specific mutants have been produced in *E*.*coli* and characterized [9, 10] [3, 11–13]. F119L has been observed in homozygous patients, but is by far more common in association with R141H [14] [15] [16, 17].

Preparations containing a single mutant do not reproduce the real proteins found *in vivo* because most patients are compound heterozygous with two different mutant alleles. F119L/R141H is the most common genotype. It accounts for 27% of patients worldwide, but in some populations this figure raises up to 86% [15] [16]. In this paper we compared for the first time the properties of F119L/R141H to those of wt/R141H. In other words we compared the enzyme found in compound heterozygous patients with the enzyme present in the cells of un-symptomatic carriers. Unexpectedly *in vitro* the specific activities of F119L/R141H and wt/R141H are similar whereas their relative stabilities are different. This finding opens the possibility for a therapeutic intervention that acts on enzyme stabilization by pharmacological chaperoning or modulation of proteostasis.

## Methods and Materials

Phosphoglucose isomerase from rabbit muscle (commercially available from Sigma Aldrich code P9544-1KU), phosphomannose isomerase from *E*. *coli*, glucose-6-phosphate dehydrogenase from bakers’ yeast (*S*. *cerevisiae*), phosphoglucomutase from rabbit muscle (commercially available from Sigma Aldrich code P3397-1KU), α-D-glucose 1,6-bisphosphate potassium salt hydrate, α-D-glucose 1-phosphate disodium salt hydrate, and β-nicotinamide adenine dinucleotide phosphate sodium salt were purchased from Sigma-Aldrich. α-D-mannose 1,6-bisphosphate was prepared and purified as described [18]. SYPRO Orange was from Invitrogen Molecular Probes. All other reagents were of analytical grade.

### Protein expression and purification and enzymatic assays

wt-PMM2, F119L and R141H were inserted into Pet22b+ generating wt-PMM2-Pet22b+, F119L-Pet22b+, R141H-Pet22b+ and expressed in *E*. *coli* BL21(DE3) strain grown at 37°C in LB broth containing ampicillin 0.2 mg/ml. The expression and purification of wt-PMM2, F119L and R141H was performed as described [1] [3] [6], with only minor changes. The XbaI/BamHI fragment of wt-PMM2-Pet22b+ or F119L-Pet22b+ was inserted into the NheI/ BamHI site of an eukaryotic expression vector vector, p.IRES2-EGFP, to obtain wt-PMM2-IRES2-EGFP and F119L-IRES2-EGFP. Cos7 cells, were cultured in DMEM with 10% FBS, 1% penicillin/streptomycin and 1% glutamine. Cells were transiently transfected with WT-PMM2-IRES2-EGFP vector (control) or with F119L-IRES2-EGFP using Lipofectamine LTX transfection reagent (Invitrogen), according to the manufacturer's instructions. 72 hours after transfection, the cells were harvested, lysed and analyzed by western blot.

PMM2 has three enzymatic activities:

$$\text{hexose}-1,6-{\text{P}}_{2}+\text{ PMM}\;=\text{ hexose}-6\text{P }\left(\text{or hexose}-1\text{P}\right)\;+\text{ PMM}-\text{P}$$ (1)

$$\text{hexose}-1\text{P }\left(\text{or hexose}-6\text{P}\right)\;+\text{ PMM}-\text{P }=\text{ hexose}-1,6-{\text{P}}_{2}+\text{ PMM}$$ (2)

$$\text{PMM}-\text{P }+{\text{ H}}_{2}\text{O }=\text{ PMM }+{\text{ P}}_{i}$$ (3)

Phosphomannomutase activity: eq (1) + eq (2), hexose = mannose

Phosphoglucomutase activity: eq (1) + eq (2), hexose = glucose

Phosphatase activity: eq (1) + eq (3), hexose = mannose or glucose

Phosphoglucomutase activity of PMM2 was assayed spectrophotometrically at 340 nm and 32°C by following the reduction of NADP+ to NADPH in 0.3 ml reaction mixture containing Hepes 20 mM, pH 7.5, MgCl_2_ 1 mM, NaCl 150 mM, NADP+ 0.25 mM, BSA 0.1 mg/ml, in the presence of 40 μM glucose 1-phosphate (Glc-1-P), 0.01 mg/ml yeast glucose 6-phosphate dehydrogenase, and 5 μM glucose 1,6-bisphosphate (Glc-1,6-P_2_). When necessary, a different concentration of Glc-1-P or Glc-1,6-P_2_ was used. In particular: i) when the effect of Glc-1,6-P_2_ concentration on the phosphoglucomutase activity was evaluated, the phosphoglucomutase activity was measured in the presence of 40 μM Glc-1P, in addition a fixed protein concentration was used (3.7 μg/ml for F119L, 2 μg/ml for wt and 6.8 μg/ml for the mixture of F119L and R141H 1: 3.5); ii) when the *K*_m_ for Glc-1-P were determined, the concentration of Glc-1,6-P_2_ was 80 μM.

Phosphomannomutase activity in fibroblasts was also measured similarly and as essentially described by Van Schaftingen et al [19] in the presence of 0.1 mM Mannose-1-phosphate and 1 μM Mannose-1,6-bisphosphate. Phosphatase activity was measured similarly using essentially the same conditions as described above. The only differences were the absence of Glc-1-P and the presence of 80 μM Glc-1,6-P2.

### Liquid chromatography coupled to electrospray mass spectrometry (RP-HPLC-ESI-MS) and size exclusion chromatography

Wild type PMM2, F119L and R141H mutants were individually diluted at 2 μM in Hepes 20 mM, NaCl 150 mM, MgCl_2_ 1 mM (pH 7.5) at 37°C for 30 min. Half of each mixture was incubated with Glc-1,6-P_2_ at 100 μM for 60 min. All samples were then analyzed by RP-HPLC-ESI-MS carried out on a Q-ToF-Premiere (Waters, Co.) equipped with Alliance binary pump using a Jupiter C4 column (5 μm, 300A°, 50 mm, Phenomenex). The chromatographic runs were carried out using H_2_O 0.1% Trifluoroacetic acid (buffer A) and acetonitrile 0.1% Trifluoroacetic acid (buffer B), from 10% to 80% of buffer B in 30 min. All mass spectra were acquired from 500 to 2500 m/z values. Horse heart myoglobin was used for tuning Q-ToF instrument and for mass calibration. All data, including ESI-MS on F119L/R141H heterodimers, were acquired and analyzed by MassLynk 4.0.

Analytical size exclusion chromatography was performed using a BioSep-SEC-S3000 column (Phenomenex) equilibrated in Hepes 20 mM pH 7.5, NaCl 150 mM, MgCl_2_ 5 mM. Wild type PMM2 (10 μg), F119L (7 μg) or a mixture of them (5 μg of wt-PMM2 and 3.5 μg of F119L) were analyzed. The chromatography was run at room temperature at 0.5 ml/min on a HPLC system by Shimadzu.

Larger-scale size exclusion chromatography was performed on a Superdex-75 column (1.5x90cm) equilibrated in the same buffer. The chromatography was run at room temperature at 0.7 ml/min on an Akta-prime system. Protein samples (0.5-1mg) (wt-PMM2, F119L, wt plus R141H (1:4.5), F119L plus R141H (1:1 or 1:3.5)) were pre-treated for 30 minutes on ice with EDTA (4 mM final concentration) and then fractionated. Fractions (2 ml each) were analyzed for protein content and enzyme activity.

### FRET assay

Alexa Fluor 488 carboxylic acid, succinimidyl ester (AF488) and Alexa Fluor 555 carboxylic acid, succinimidyl ester (AF555), were from Molecular Probes. We performed the conjugation of AF488 with F119L and that of AF555 with R141H following the manufacturer’s instructions. Protein samples, 1 mg of each protein (2 mg/ml in PBS pH 7.2), were treated with 50 μl sodium bicarbonate 1M and added to the reactive dye. The reaction was conducted for 1.5 h at room temperature under gentle stirring. Eventually the unreacted dye was removed by gel filtration.

AF488-F119L and AF555-R141H (3.9 and 6.75 μg respectively) were mixed (in 400 μl Hepes 20 mM pH 7.5, NaCl 150 mM, MgCl_2_ 1 mM). An equal sample was treated for 24h at 4°C with thermolysin (1.5 μg/ml in the presence of CaCl_2_ 1.25 mM). The experiment was repeated three times under identical conditions.

We conducted FRET measurements following the experimental procedure described by Chakraborty *et al* [20] based on the theoretical and experimental procedure developed by Song *et al* [21].

The FRET signal was calculated using the equation E_m FRET_ = E_m total_−*x*F_(D)_−*y*F_(A)_

From measurements of different concentrations of pure AF488-F119L or AF555-R141H, we determined *x* = 0.203±0.008 and *y* = 0.088±0.001.

Fluorescence was measured at room temperature by using a Cary Eclipse Spectrofluorimeter.

### Assessment of stability *in vitro* and *in vivo*

Heat-induced melting profiles of wt-PMM2, F119L and R141H were recorded by thermal shift assay or by circular dichroism. In both cases the proteins (0.2 mg/ml) were equilibrated in Hepes 20 mM pH 7.5, MgCl_2_ 1 mM, NaCl 150 mM, DTT 1mM, and the temperature was increased at 0.5°C/min.

Thermal shift assay was performed by using the iCycler iQ Real Time PCR Detection System (Bio-Rad Laboratories, Hercules, CA-USA). The proteins were heated from 25 to 80° in the presence of Sypro Orange 2.4x (Invitrogen Molecular Probes). The normalized melting profiles were obtained using the equation fu(T) = f(T)-fn/fd-fn, where fn represents the minimum value of the fluorescence before the transition and fd represents the maximum value after the transition [22].

When the melting profile was obtained by circular dichroism (CD) (Jasco J-815 Circular Dichroism Spectrometer), the signal of the protein samples at 220 nm was recorded in the range 20–70°C.

The unfolded fraction was calculated as fu(T) = f(T)—fn(T)/fd(T)—fn(T) where f is the CD ellipticity at 220 nm at temperature T, fn(T), and fd(T) are the values of ellipticity extrapolated at temperature T from the native and unfolded regions of the melting profile.

For limited proteolysis the proteins (wt-PMM2, F119L, R141H, 0.38 mg/ml in Hepes 20 mM pH 7.5, MgCl_2_ 1 mM, NaCl 150 mM) were treated with trypsin (1:50 protease:enzyme ratio) at 37°C [23].

Urea-induced unfolding of wt-PMM2, F119L and R141H (0.275 mg/ml, Hepes 20 mM pH 7.5, MgCl_2_ 1 mM, NaCl 150 mM) were performed by incubating the pure proteins in the presence of increasing concentration of urea in the presence of Sypro Orange 4x. Fluorescence was recorded at 580 nm (excitation at 485 nm) after 2 h incubation at room temperature. Data were plotted as fraction of protein unfolded. Urea-induced unfolding of PMM2s (wt, F119L, wt/R141H 1:6, and F119L/R141H 1:5 recovered after gel-filtration fractionation) were determined by recording the residual enzymatic activity. The proteins (0.2 mg/ml, Hepes 20 mM pH 7.5, MgCl_2_ 1 mM, NaCl 150 mM) were incubated for 2 hours at 10°C after which the residual phosphoglucomutase activity was measured under standard condition. The reaction mixture (containing appropriate amount of protein) was incubated for known interval times (20–40min), then the reaction was stopped by addition of sodium carbonate 1M and the fluorescence of the NADPH produced was recorded (excitation 340nm, emission 445nm). Data were plotted as normalized residual activity, i.e. the ratio between the activity measured at a given concentration of denaturant divided by the activity in the absence of denaturant. Fluorescence was measured by using a Cary Eclipse Spectrofluorimeter equipped with a high throughput microplate reader.

For western blot analysis polyclonal antibody (10666-1-AP Proteintech) for the detection of PMM2 were used. Adequate amounts of proteins (10 μg) were subjected to SDS-PAGE (15%) and were transferred to PVDF membrane. The detection was performed by using the Immun-Star WesternC chemiluminescence detection kit (Bio-Rad).

Fibroblasts were a gift from Prof. Flemming Skovby (rigshospitalet.dk) who had obtained with informed consent from patients in accordance with The Code of Ethics of the World Medical Association (Declaration of Helsinki) [11]. Cells were grown in RPMI medium supplemented with 2 mM glutamine, 10% FCS and 100units/ml penicillin/streptomycin until confluency. Two cycles of freeze/thaw were used to prepare cell lysate in Hepes 20 mM pH 7.5, NaCl 150 mM, MgCl_2_ 1 mM in the presence of protease inhibitor cocktail (P8340, Sigma). Clear extracts were then obtained by centrifugation.

## Results

### Wild type PMM2, hypomorphic F119L and inactive R141H are phosphorylated by glucose-1,6 bis-phosphate

**Fig 1:**
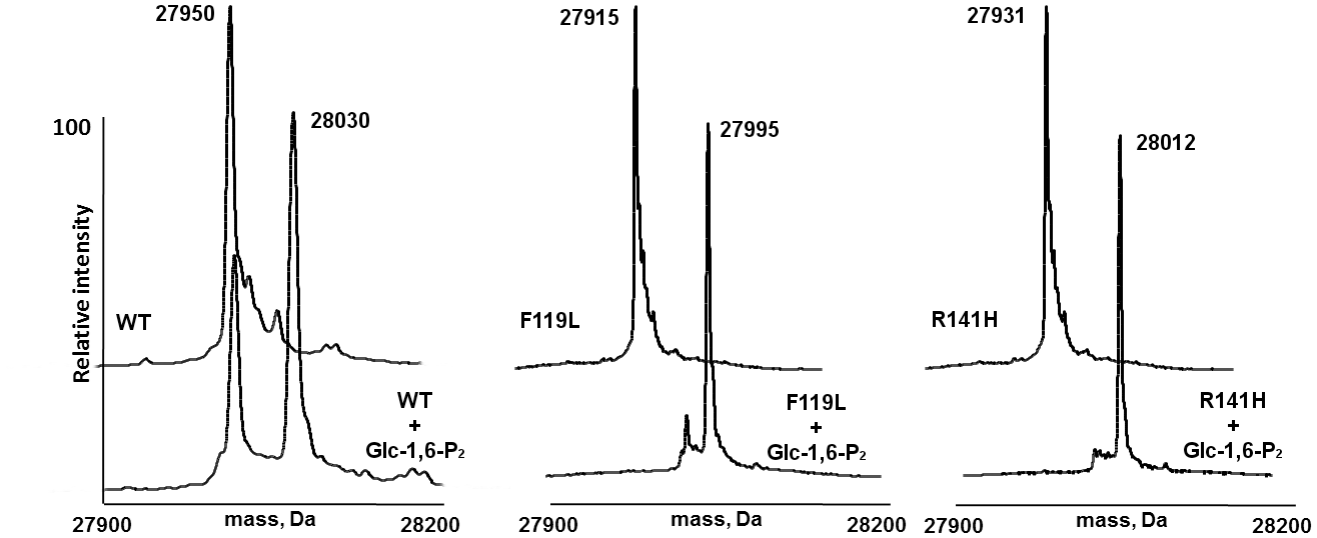
Mass spectrometry analysis of wild type phosphomannomutase2, F119L and R141H, with and without Glucose-1,6-bisphosphate. Panel A shows the deconvoluted mass spectrum of wt-PMM2 (2 μM) upon RP-HPLC-ESI-MS analysis of the incubation mixture in absence (back peaks) and in presence of Glc-1,6-P_2_ (250 μM, front peaks), showing an increment of 80 Da attributed to a single phosphorylation event. Panels B and C show the same experiment carried out on F119L and R141H, respectively.

Phosphomannomutase and phosphoglucomutase activity of wild type and F119L have already been compared [3]. F119L has an activity that varies from half to one third of that of the wild type, depending on the condition of the assay. Homogeneous R141H expressed in *E*. *coli* is inactive [9–11], in fact its phosphomannomutase and phosphoglucomutase specific activities are two orders of magnitude lower than those of wild type. The specific phosphatase activity of R141H is very low, 0.004±0.001U/mg as those of wild type, 0.008±0.003 U/mg and F119L, 0.009±0.001U/mg. Although mutations prevent or slow down catalytic activity, they do not prevent the phosphorylation of the enzyme by bis-phosphate sugar activators. We incubated wild type and PMM2 mutants for 30 min in the presence of 50-fold molar excess of Glc-1,6-P_2_ and the mixtures were analyzed by RP-HPLC coupled to MS. As reported in Fig 1 (panels A, B, C), a single phosphorylation, corresponding to an increment of 80 Da, occurs on each protein.

More in detail, wt-PMM2 showed a MW of 27950±1 Da and of 28030±3 Da; F119L showed a MW of 27915±1 Da and of 27995±2 Da, R141H showed a MW of 27931±4 Da and of 28012±2 Da, each pair without and with Glc-1,6-P_2_, respectively. In a previous paper by us, it was further proved that Glc-1,6-P_2_ induces a conformational transition in wild type as well as on mutant PMM2 proteins. Therefore, although abortive, the mechanism of phosphorylation and domain closure, which occurs in the wild type enzyme, takes place also in the completely inactive mutant R141H [6].

### Specific activity of F119L is enhanced in the presence of R141H

**Fig 2:**
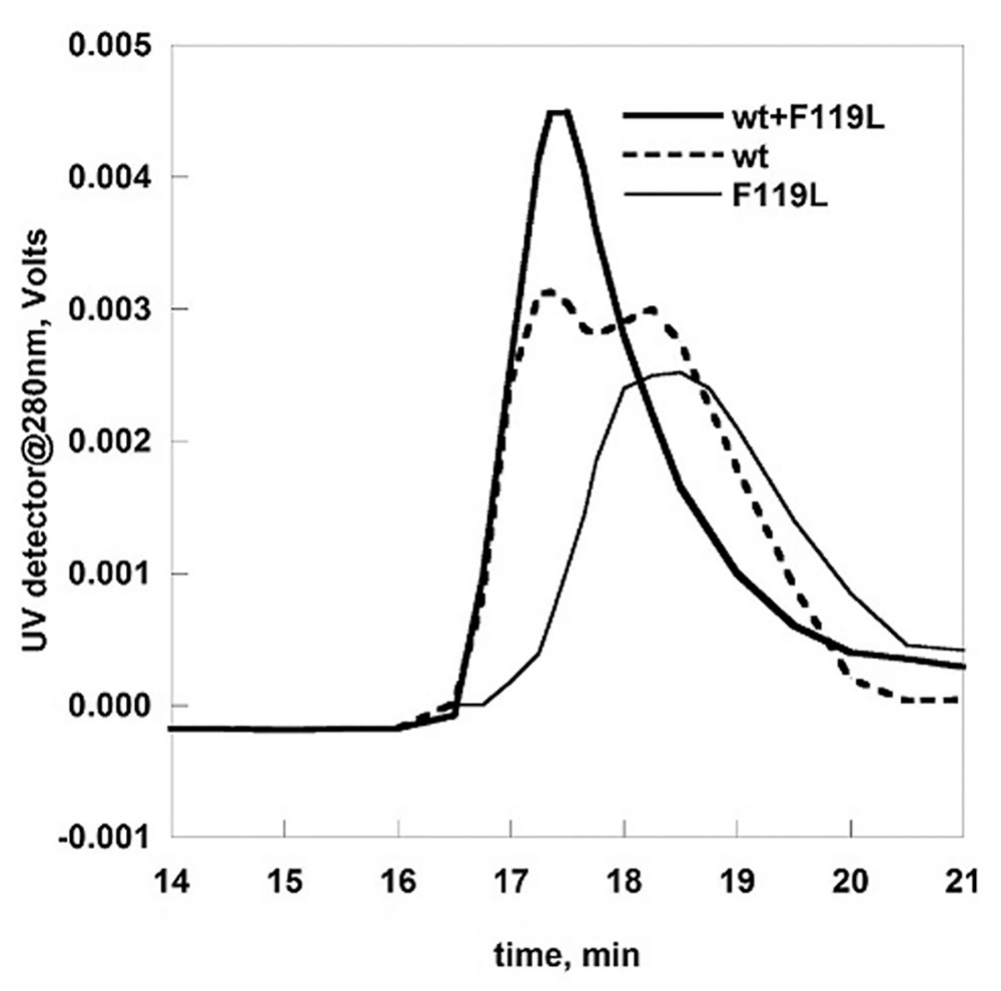
Analytical gel filtration analysis of wild type and F119L phosphomannomutase2. wt-PMM2 (10 μg), F119L (7μg) and a mixture of them (5 μg of wt-PMM2 and 3.5 μg of F119L) were analyzed by gel filtration on BioSep-SEC-S3000 column at 0.5 ml/min in Hepes 20 mM pH 7.5, NaCl 150 mM, MgCl_2_ 5 mM.

We analyzed the quaternary structure of wt-PMM2 (Fig 2 dashed line) and F119L (Fig 2 thin continuous line) by size-exclusion chromatography. The equilibrium between monomer and dimer is shifted towards the monomer in the case of the mutant.

This result is not unexpected and is consistent with our previous study where we had determined the molecular mass of the wt-PMM2 and F119L under native conditions, 52000±200 and 34000±700, by Light-scattering [3]. We carried out a different experiment in which similar amounts of the two proteins were mixed before being loaded onto a size-exclusion column. Unexpectedly we could observe a single peak with the elution volume of the dimer (Fig 2 thick continuous line).

One possibility could be that a heterodimer forms. The mutation F119L affects dimerization when present on both subunits of a homodimer F119L/F119L, but has a less damaging effect when present on a single subunit of a complex wt/F119L. This observation is, in our opinion, *per se*, interesting. Nonetheless we are aware that a single mutated allele is not sufficient to cause disease and for this reason, a mixed dimer wt/F119L is not a target for drug development. For this reason we decided to move to a case which is associated to a pathologic state in humans. So far the most common genotype encountered in PMM2-CDG patients is F119L/R141H. The mutation R141H occurs in the active site, far from the region that is engaged in protein-protein interaction. Therefore R141H should provide a surface for subunit assembly as “healthy” as that of wt-PMM2.

**Fig 3:**
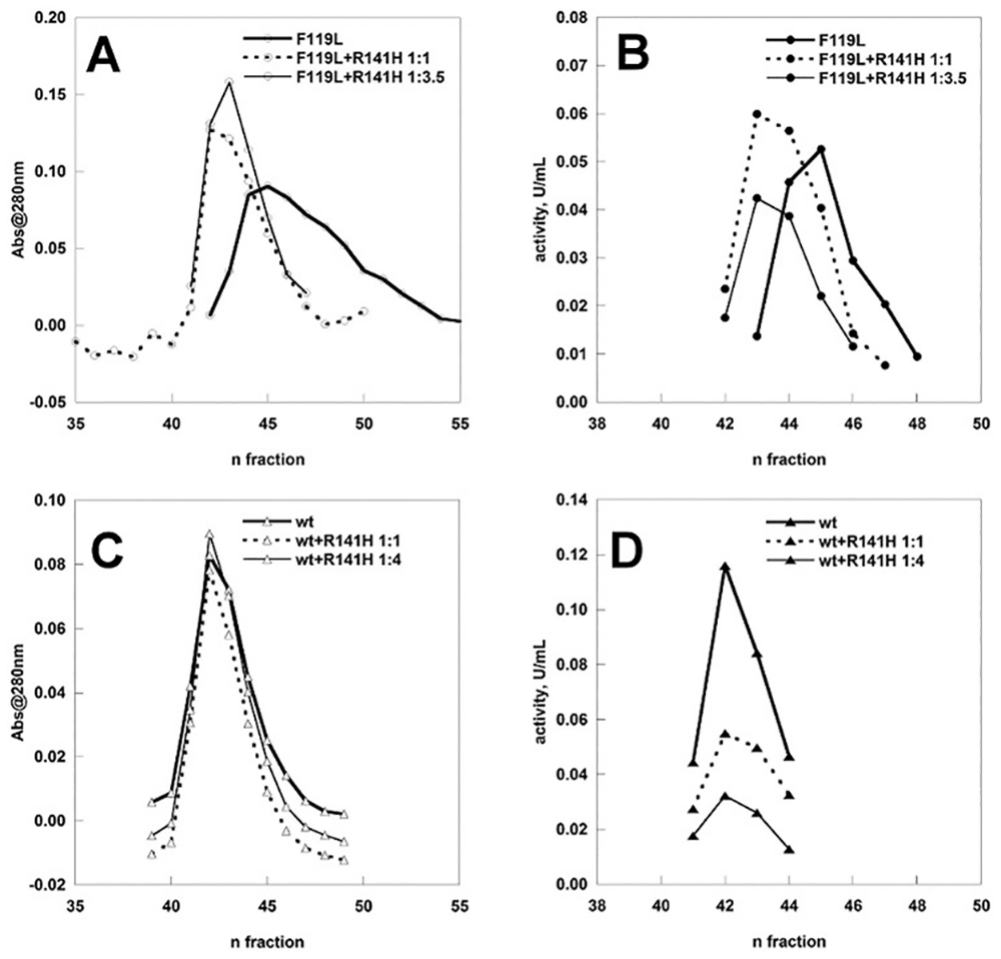
Preparative gel filtration of wild type and F119L phosphomannomutase2. wt-PMM2 and F119L, alone and in the presence of R141H were fractionated on a Superdex75 column equilibrated in Hepes 20 mM pH 7.5, NaCl 150 mM, MgCl_2_ 1 mM. The fractions were analyzed for the protein content and for the phosphoglucomutase activity (in the presence of 40 μM Glc-1P and 5 μM of Glc-1,6-P_2_). Before loading, each protein sample was pretreated with EDTA on ice. Panel A and B: F119L and F119L+R141H (1:1 and 1:3.5). Panel C and D: wt-PMM2 and wt-PMM2+R141H (1:4).

We produced R141H and F119L separately in *E*.*coli*, and we mixed the two purified proteins in equimolar amounts to replicate *in vitro* the composite heterozygous phenotype. In order to induce the dissociation of any homodimeric species, the proteins were mixed in the presence of EDTA [3]. The mixture was loaded onto a Superdex 75 column. The eluent buffer was Hepes 20 mM pH 7.5, containing NaCl 150 mM and MgCl_2_ 1 mM, the divalent ion required for the assembly of subunits. As a control an equal amount of F119L was subjected to the same treatment. As shown in Fig 3 panel A the peak of F119L (dashed line) in the presence of R141H is sharper than that of F119L alone (thick continuous line) and is eluted faster. This behavior can be explained assuming that R141H induces F119L to associate and form heterodimers. If a molar excess (3.5:1) of R141H over F119L is employed, the peak is eluted even sharper (thin continuous line) suggesting that the equilibrium between different quaternary structures can be further shifted towards the formation of dimers.

**Fig 4:**
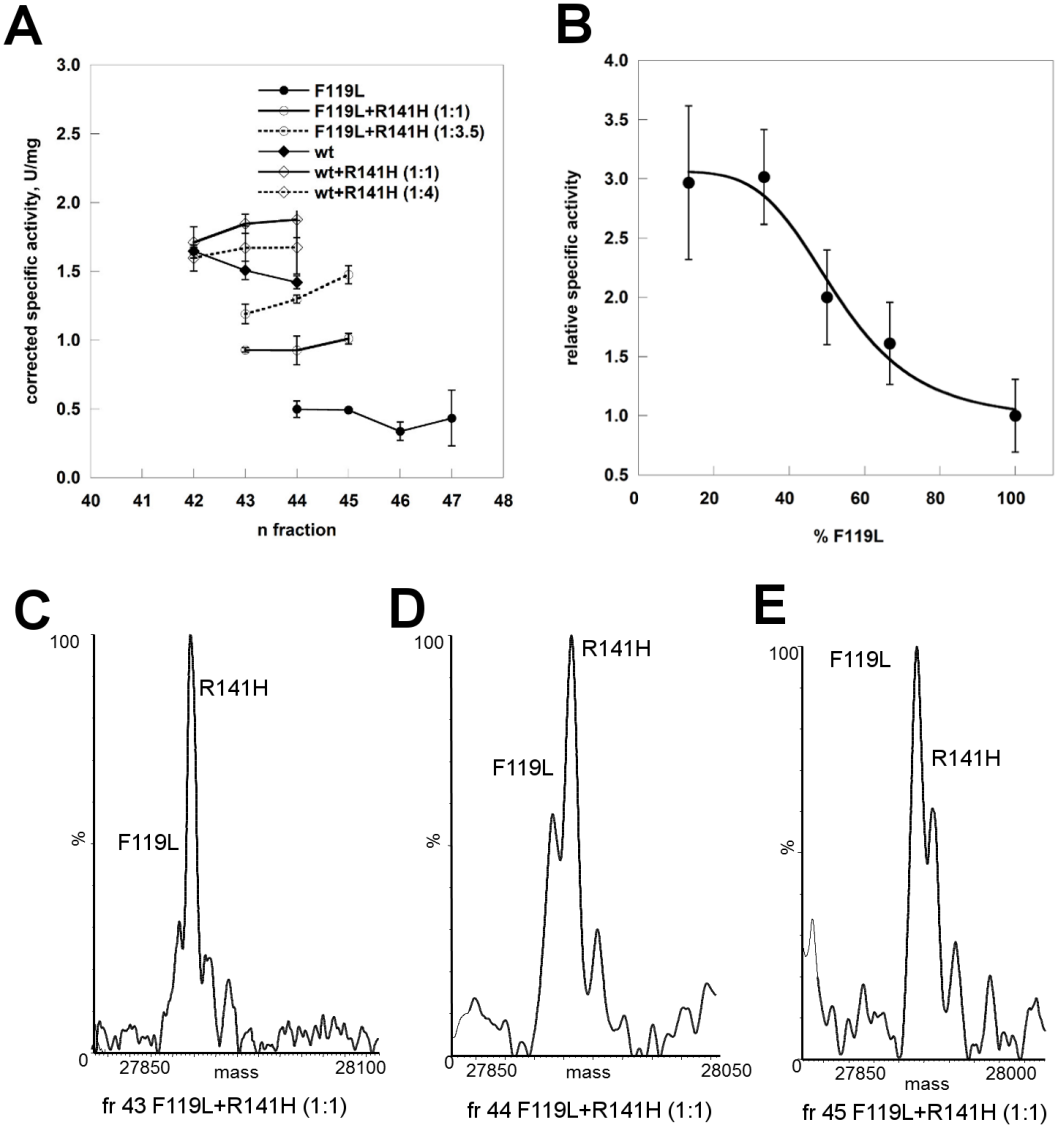
Specific activity analysis of phosphomannomutase2. Panel A) The samples (wt-PMM2, F119L, wt-PMM2 plus R141H (1:1 or 1:4), F119L plus R141H (1:1 or 1:3.5) were fractionated on a Superdex75 column. The specific protein ratio of the sample loaded was taken into account in order to calculate the corrected specific activity shown. The protein content of F119L plus R141H (1:1) fr.43,44 and 45 were analyzed by ESI-MS and the deconvoluted mass spectra are shown in panel C, D and E respectively. Panel B) F119L, F119L plus R141H (1:0.5; 1:1; 1:2; 1:6.5) were analyzed in batch and the activity expressed as fold increase relative to pure F119L. The phosphoglucomutase activity was measured in the presence of 40 μM Glc-1P and 5 μM of Glc-1,6-P_2_.

The fractions eluted from the column were assayed to test the effect of the inactive subunit on the activity of F119L (Fig 4 panel B). It is worth remembering that although the three samples subjected to size exclusion chromatography contained the same total amount of proteins, the proportion of the only active species, F119L, was different and represented either 100%, 50% or 22% of the total loaded protein. Nonetheless the activity profiles do not decline accordingly.

We carried out a control experiment in which wt-PMM2 was analyzed either alone or in mixture, 1:1 or 1:4, with R141H. The total amount of proteins loaded on the column, was the same. The eluted fractions were assayed and the chromatograms and the enzymatic activity (U/ml) are reported in Fig 3 panel C and D respectively. In this case we observed that the activity declines as the percentage of wt-PMM2 diminishes in the sample loaded. Each experiment was repeated at least three times with consistent results.

In Fig 4, Panel A the corrected specific activity was calculated dividing the enzymatic units measured in the fractions by the concentration of active form, wt-PMM2 or F119L, i.e. 100% (graphs with filled diamonds or circles) or, 50% (graph with empty diamonds or circles and continuous lines), 22% (graph with empty circles and dashed lines) or 20% (graph with empty diamonds and dashed lines), of total mg loaded. Under the experimental condition employed, 5 μM Glc-1,6-P_2_ and 40 μM Glc-1P, the corrected specific activity doubles when the ratio between the hypomorphic and the inactive mutant is 1:1 and is 3 fold higher when the ratio is 1:3.5 compared with that of F119L alone. On the other hand the corrected specific activity of the wt-PMM2 is not influenced by the addition of the inactive mutant. We assayed the composition of the fractions 43, 44, 45 eluted after loading F119L+R141H (1:1) by electrospray mass spectrometry (ESI-MS) (Fig 4, Panel C, D and E).

In order to confirm the fact that R141H enhances the specific activity of F119L, we carried out an experiment in batch where we can control the relative concentration of the two species. We prepared 5 samples containing F119L 0.18 mg (100%), F119L 0.12 mg/R141H 0.06 mg (1:0.5; 66%), F119L 0.09 mg/R141H 0.09 mg (1:1; 50%), F119L 0.06 mg/R141H 0.12 mg (1:2; 33%), F119L 0.024 mg/R141H 0.156 mg (1:6.5; 13%) as previously described and let them equilibrate for 30 min on ice then dialyzed, before assaying the phosphoglucomutase activity. Specific activity was calculated dividing enzymatic units by the amount of F119L. The results are reported in Fig 4 panel B as the fold increase relative to the sample containing only F119L. The enhancing effect reaches a plateau when the ratio between the hypomorphic and the inactive mutant is 1:2 and the corrected specific activity is comparable to that of wt-PMM2. Since monomeric PMM2 is inactive [3] and we can assume that the enzymatic activity is proportional to the amount of dimeric species, F119L is present either as a homodimer or an heterodimer when the plateau is reached, i.e. when the ratio between the two mutants is 1:2.

### Förster/Fluorescence resonance energy transfer (FRET) assay shows the formation of F119L/R141H heterodimers

**Fig 5:**
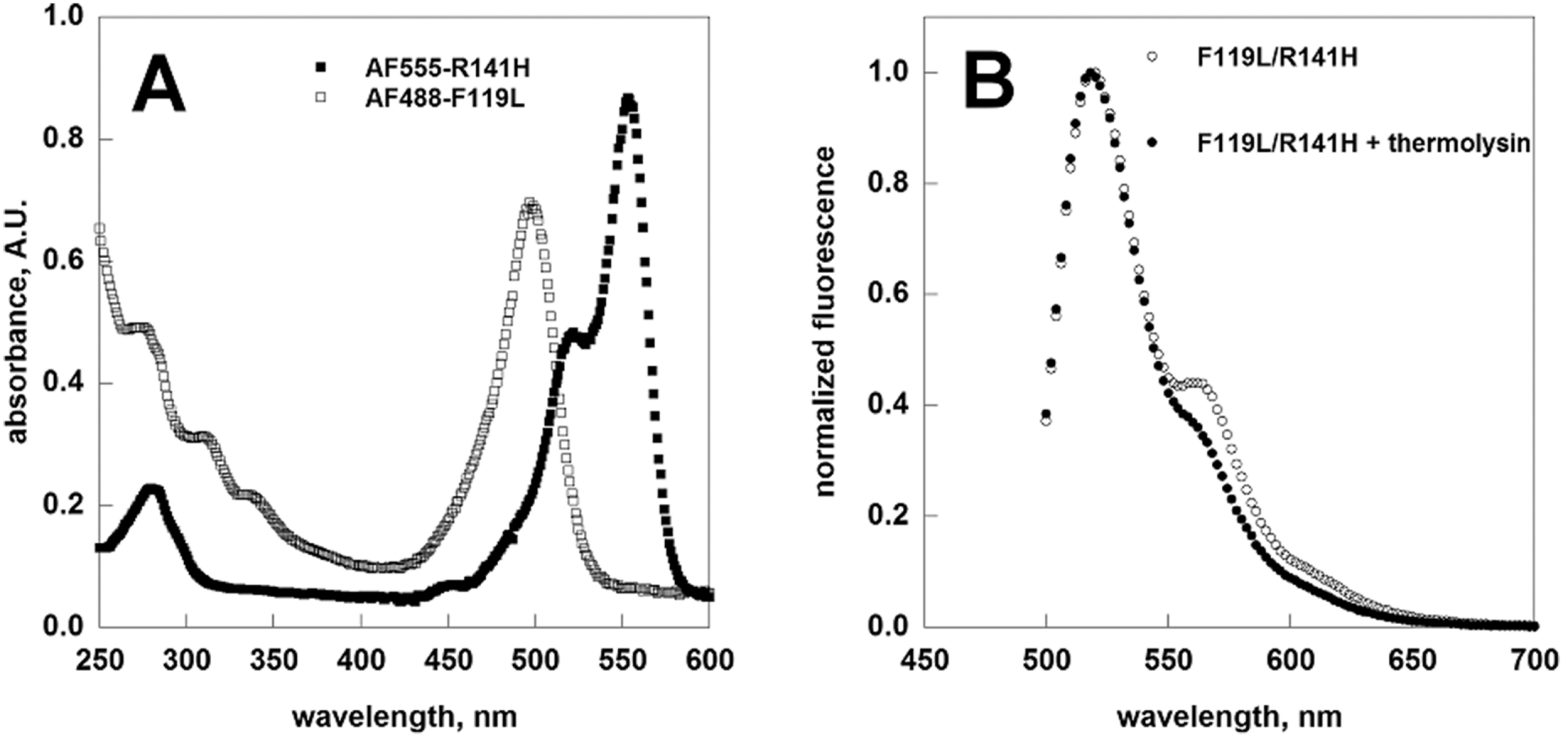
FRET measurements. The dye-protein conjugates (AF555-R141H 0.15 mg/ml, AF488-F119L 0.26 mg/ml) were analyzed by UV-spectroscopy (Panel A). Normalized fluorescence emission spectra of a mixture of AF488-F119L (9.75 μg/ml) and AF555-R141H1 (16.9 μg/ml) recorded upon excitation of 470nm is shown (Panel B, emply circles). The emission spectra obtained when the protein mixture was pre-treated with thermolysin is showed for comparison (Panel B, filled circles).

F119L and R141H were covalently labelled with Alexa Fluor 488 (λex = 495/λem = 519) and Alexa Fluor 555 (λex = 555/λem = 565) respectively. AF448-F119L served as the donor and AF555-R141H as the acceptor. Pure proteins were analyzed by UV-visible spectroscopy (Fig 5, panel A empty and filled squares). The fluorophore to monomer protein (F/P) molar ratio was obtained for each conjugate. Based on UV-visible absorption spectra the F/P were calculated to be 0.94 for AF488-F119L and 1.01 for AF555-R141H.

AF488-F119L and AF555-R141H (3.9 and 6.75 μg respectively) were then mixed in Hepes 20 mM pH 7.5, NaCl 150 mM, MgCl_2_ 1 mM. As a negative control, an equal sample was treated for 24h at 4°C with thermolysin (1.5 μg/ml in the presence of CaCl_2_ 1.25 mM). The spectrum recorded under this condition represents the emission of AF488 and AF555 when no energy transfer due to protein-protein interaction is possible. The protein mixtures were excited at 470 nm and their emission spectrum was recorded (Fig 5, panel B). After subtracting the contribution of direct emissions of the donor and of the acceptor (see Methods), the absolute FRET signal at 570 nm was found equal to 2.31±0.10.

### The activity of F119L/R141H heterodimers is enhanced by glucose1,6-bisphosphate

We measured the *K*_m_ for Glc-1-P in the presence of 80 μM Glc-1,6-P_2_ for pure samples and for the heterodimers prepared by gel-filtration, wt/R141H (1:6) or F119L/R141H (1:4.5). The large excess of R141H should ensure that the presence of active homodimers or monomers is negligible. The affinity of wt-PMM2 for the substrate is not affected by R141H (*K*_m_ is 3.6±0.9 μM or 3.1±1.2 μM in the presence of a large excess of R141H) whereas that of F119L is increased in the presence of the inactive mutant (*K*_m_ is 45.2±11.8 μM or 17.1±7.6 μM in the presence of a large excess of R141H).

**Fig 6:**
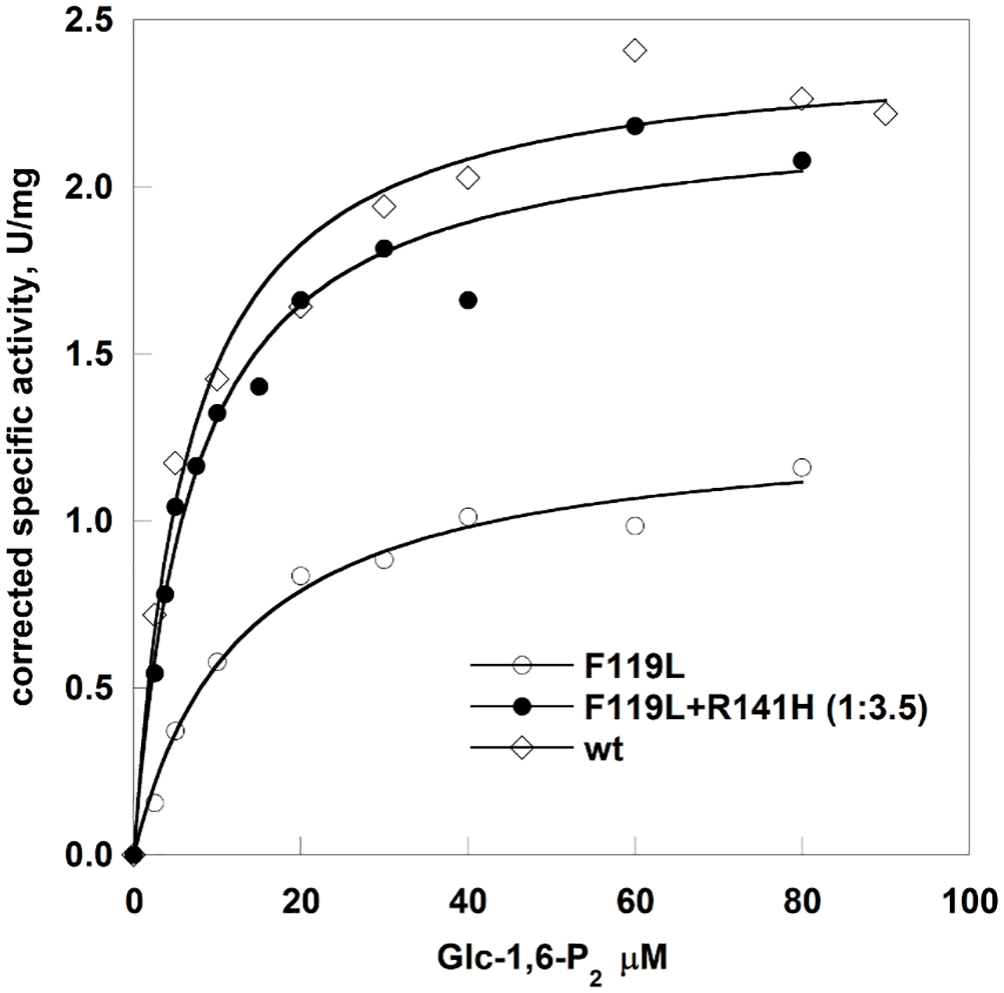
Effect of glucose1,6-bisphosphate concentration on the phosphoglucomutase activity of wild type phosphomannomutase2, F119L and F119L+R141H. Protein samples from the Superdex75 fractionations were used. The phosphoglucomutase activity was measured in the presence of 40 μM Glc-1P. The specific protein ratio of the sample F119L+R141H was taken into account in order to calculate the corrected specific activity.

The activity of PMM2 is greatly influenced by the bis-phosphate activators and the relative activity of F119L (open circles in Fig 6) and wt-PMM2 (open diamonds in Fig 6) depends on the concentration of Glc-1,6-P_2_. We measured the activity of the proteins eluted from the gel filtration as a function of Glc-1,6-P_2_. In particular we assayed three samples, F119L/R141H (1:3.5) besides wt-PMM2 and F119L.

We carried out experiments using variable concentrations of the activator Glc-1,6-P_2_ at fixed protein concentration, F119L, wt-PMM2 and the mixture of F119L and R141H (1:3.5) and at fixed substrate concentration, 40 μM Glc-1-P. Corrected specific activity was calculated per microgram of active subunit. This means that the specific activity of the mixture (filled circles line in Fig 6) is obtained dividing the enzymatic units per ml by the protein concentration and by 0.22 (percentage of F119L in the mixture). The hyperbolic dependence of velocity on the activator concentration was fitted using Michaelis and Menten equation to evaluate the concentration at which Glc-1,6-P_2_ exerts half of its maximal effect. The EC50 for F119L (12.7±1.80 μM) is higher than that for wt-PMM2 (6.51±1.02 μM), but in the presence of R141H they become comparable (7.04±0.96 μM).

### Mutant forms of phosphomannomutase2 are unstable *in vitro* and *in vivo*

**Fig 7:**
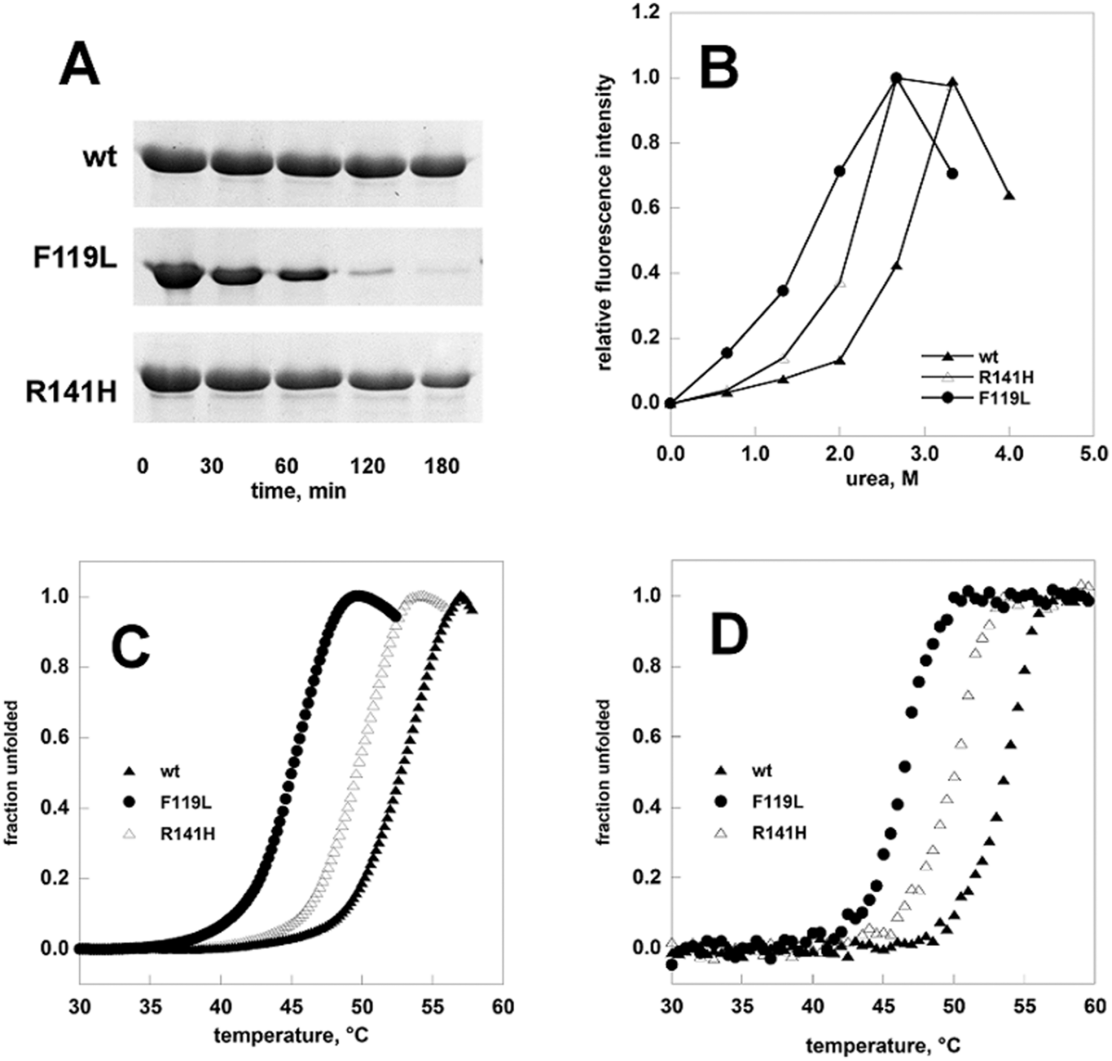
Comparison of the stabilities of phosphomannomutase2. Panel A: wt-PMM2, F119L, and R141H were treated with trypsin (1:50 protease:enzyme ratio) and the time-course of the reaction was monitored by SDS-PAGE. Panel B: wt-PMM2, F119L, and R141H (0.275 mg/ml) were incubated in the presence of increasing concentration of urea. The incubation was conducted at room temperature in the presence of Sypro Orange 4x, the emitted fluorescence was recorded and data were normalized. Panel C: temperature melting profiles of wt-PMM2, F119L and R141H were recorded by thermal shift assay. The proteins (0.2 mg/ml) were heated from 25 to 80° at 0.5°C/min in the presence of Sypro Orange 2.4x. Panel D: temperature melting profiles of wt-PMM2, F119L and R141H recorded by circular dichroism. The proteins (0.2 mg/ml) were heated from 20 to 70° at 0.5°C/min and the signal at 220nm was recorded. All the experiments were conducted in Hepes 20 mM pH 7.5, NaCl 150 mM, MgCl_2_ 1 mM.

We tested the stability of F119L and R141H by limited proteolysis, urea induced or thermal induced denaturation and compared it to that of the wild type enzyme (Fig 7). The results obtained by any technique show that the hypomorphic mutant is the less stable form and that the inactive mutant has intermediate stability.

**Fig 8:**
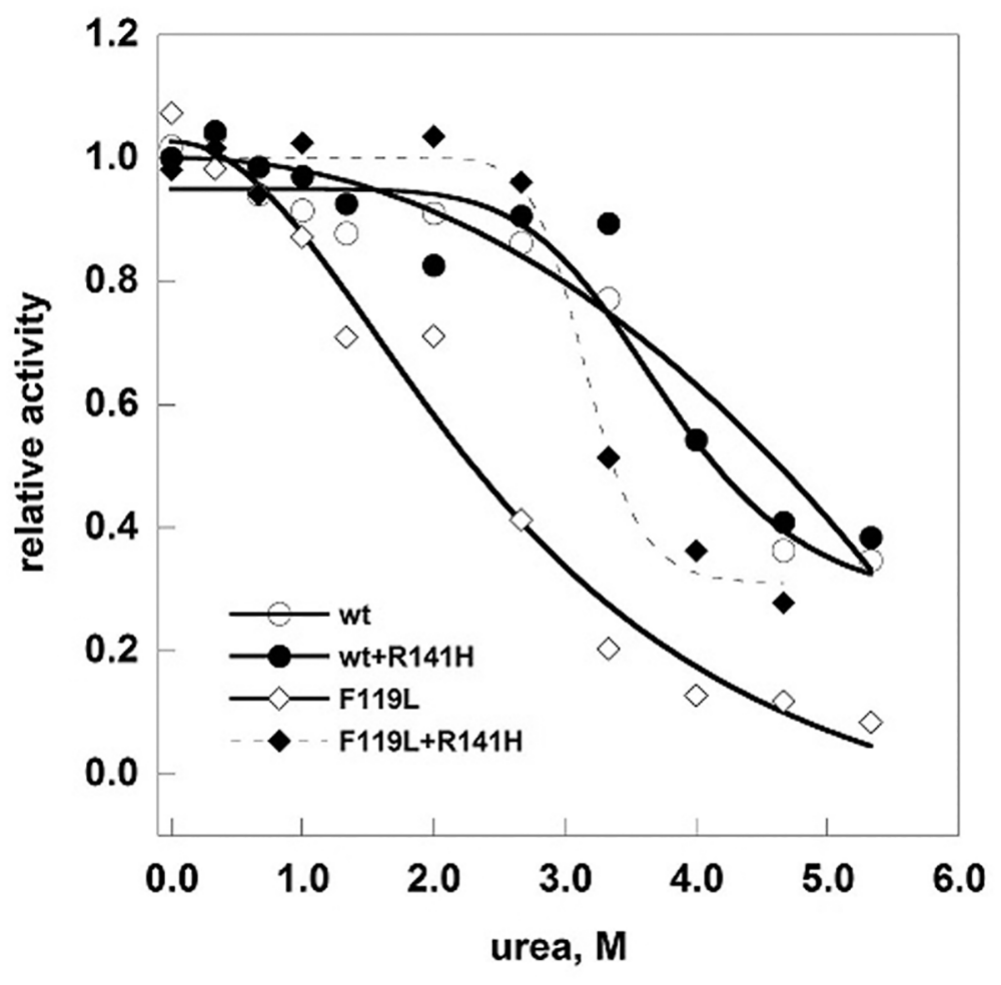
Urea-induced melting profile of phosphomannomutases2. The proteins (wt-PMM2, F119L, wt/R141H 1:6, and F119L/R141H 1:5, 0.2 mg/ml in Hepes 20 mM pH 7.5, MgCl_2_ 1 mM, NaCl 150 mM) were equilibrated with urea (from 0 to 6 M) for 2 hours at 10°C after which the residual phosphoglucomutase activity was measured under standard condition. Data were expressed as residual enzymatic activity.

In order to test the stability of the mixed proteins we determined the melting curves in the presence of a denaturant by following the enzymatic activity (Fig 8). We challenged either pure proteins, wild type or F119L, or mixtures, wt/R141H (1:6) or F119L/R141H (1:5) with different amounts of urea, the total protein concentration being the same (0.2 mg/ml). After attaining equilibrium, the samples were diluted in the assay buffer and residual phosphoglucomutase activity was measured. The curve of normalized residual activity, i.e. the ratio between the activity measured at a given concentration of denaturant divided by the activity in the absence of denaturant, indicates that in the presence of the inactive mutant F119L unfolds at a higher concentration of urea, but even so it is more sensitive to denaturation than the wild type either or wt/R141H.

**Fig 9:**
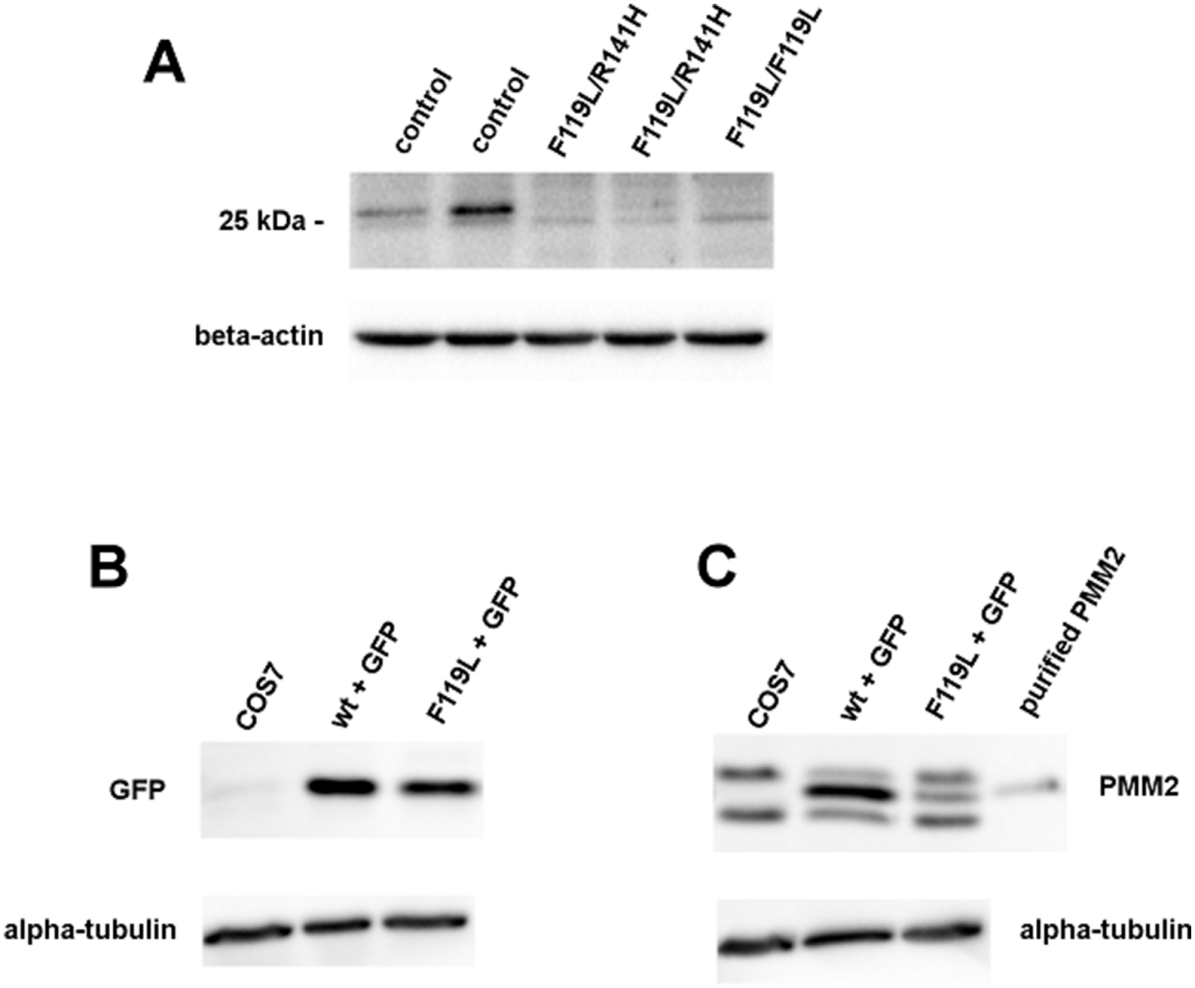
Comparison of phosphomannomutase2 contents by western-blotting analysis. Cell extracts (two healthy control fibroblasts and three primary fibroblasts of patients, 10 μg of each) were analyzed and protein were visualized by incubation with polyclonal anti-PMM2 antibody (A).Cell extracts of Cos7 transiently transfected with wt-PMM2-IRES2-EGFP vector or with F119L-IRES2-EGF (10 μg of each), were analyzed and protein were visualized by incubation with polyclonal anti-GFP antibody (B) and polyclonal anti-PMM2 antibody (C) Beta-actin (Panel A and B) or Alpha-tubulin were used as loading controls and purified wt-PMM2 as a standard (Panels B and C).

Mutants that are unstable *in vitro* tend to be unstable *in vivo* too and, as a consequence, have a lower concentration in the cell (Fig 9, Panel A). The amount of PMM2 in primary fibroblasts of patients carrying F119L/R141H (lane 3 and 4) or F119L/F119L (lane 5) genotype was detected by western blot and compared with that of healthy controls (lane 1 and 2).

As seen in Fig 9, Panel A, the amount of the protein in healthy controls can be different, but in any case it exceeds the amount found in the patients. The experiment was repeated at least three times with consistent results.

Phosphomannomutase activity was also measured in fibroblast extracts. The activity found in healthy controls was 1.70±0.45 mU/mg, whereas in patients the activity was 0.13 and 0.16 mU/mg as far as F119L/R141H is concerned (lane 3 and 4), 0.19 mU/mg in the genotype F119L/F119L (lane 5).

In order to confirm that F119L is less stable than wt-PMM2 *in vivo*, the open reading frames encoding the wild type and mutant proteins were cloned into the eukaryotic expression vector pIRES2-EGFP and transiently expressed into Cos7 cells. 72 hours after transfection, the cells were harvested, lysed and analyzed by western blotting (Fig 9, panel B and C). The vector also expresses EGFP (enhanced green fluorescent protein) which was used as a control of the transfection efficiency. To distinguish the specific band of transfected PMM2 and F119L from the two aspecific bands, we loaded the purified protein expressed in *E*.*coli* and an extract of Cos7 cells. Alpha-tubulin was used as a loading control and purified wt-PMM2 as a standard.

## Discussion

**Fig 10:**
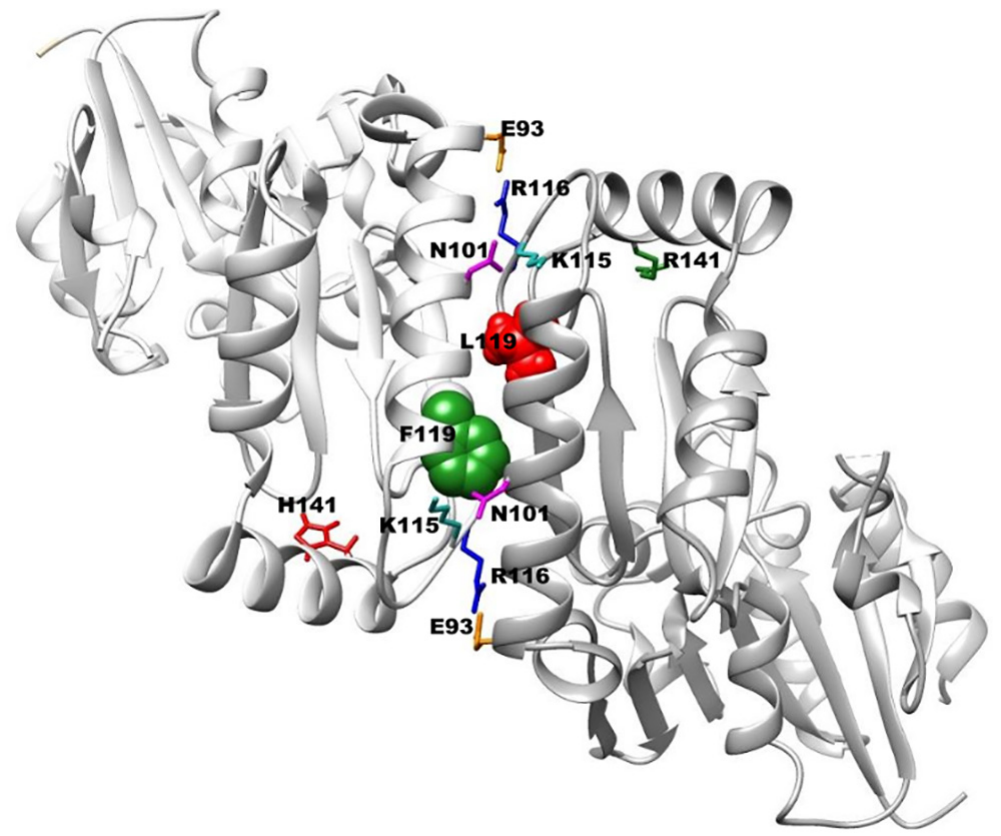
F119L/R141H structural model. The two chains are represented as cartoons in light or dark grey. F119 on one chain and L119 on the other chain, are shown as spheres in green or red respectively. R141on one chain and H141 on the other chain, are shown as sticks in green or red respectively. The side chains of E93 and R116 forming a salt bridge at the interface, are shown as sticks in orange or blue respectively. The side chains of K115 and N101 forming a H-bond at the interface, are shown as sticks cyan or magenta respectively.

In a previous paper by us we demonstrated that PMM2 works as a dimer and estimated the dissociation constant of wild type and F119L homodimers [3]. The effect of F119L mutation on the quaternary structure of the enzyme can be explained in the light of the 3D structure: the presence of bulky F119 results in a specific conformation for the loop between K115 to F119, that allows interactions of K115 and R116 with N101 and E93 of the dimeric partner. Replacement of bulky F by L, affects the hydrophobic core and very likely alters the disposition of K115 and R116 resulting in alteration of the interactions in the dimer. This effect is strong when F119L mutation is present on both subunits, but results in a loss of stability, but keeping activity, when present only in one subunit. In Fig 10 we show a possible model of the heterodimer F119L/R141H.

Disease missense mutations reduce the activity of a protein in the cell by different mechanisms: they can lower the specific activity, they can prevent folding or they can affect the stability of the folded protein. In the last case reduced activity is only a secondary effect because unstable mutant proteins have a lower lifetime, and hence a lower concentration in the cell. It is mandatory to distinguish the mechanisms by which mutations exert their deleterious effect. Only for the third case in fact, small molecule drugs can reverse the effect of the mutation by stabilizing the protein, increasing its concentration and hence restoring the activity. Could small molecule drugs, pharmacological chaperones or drugs that affect proteostasis, be used to treat PMM2-CDG as it occurs for other genetic diseases [24–26]? In order to answer this question, specific genotypes must be analyzed and we started from the most common one in PMM2-CDG.

It was demonstrated that F119L has an activity that varies from half to one third of that of the wild type, depending on the condition of the assay. As shown in Fig 10, F119 does not occur in the active site, but at the interface between the two subunits. Its effect on activity depends on the fact that only dimeric PMM2 is active. This finding would suggest that the mutation affects specific activity and for this reason it cannot be rescued by chaperones or drugs that affect proteostasis. F119L has been observed in homozygosity, but is by far more common in association with R141H. We have mixed R141H with F119L or with wild type to reproduce and compare the type of proteins found in the compound heterozygous patients or in healthy carriers. We observed that hydrodynamic properties of F119L vary in the presence of the inactive mutant suggesting the formation of a heterodimer and that the activity is similar to that observed for mixtures of wt-PMM2 and R141H. Admittedly our analysis of the quaternary structure of mixed samples is only qualitative as it would be very difficult to distinguish heterodimers from homodimers. Nonetheless we observed that the association with the common inactive mutant R141H mitigates the enzymatic defects of the hypomorphic mutant F119L.

Then why is F119L/R141H a severe genotype compared with wt/R141H? Let us over-simplify the model and assume that in the cells of the asymptomatic carriers, who carry one wild type allele and one R141H allele, 50% of the proteins are in the form of a heterodimer wt/R141H, 25% in the form R141H/R141H and 25% in the form wt/wt whereas in the cells of the patients, who carry one F119L allele and one R141H allele, 50% of the proteins are in the form of the heterodimer F119L/R141H, 25% in the form R141H/R141H and 25% in the form of the monomers F119L. Since we have shown that the specific activity of F119L/R141H and that of wt/R141H are comparable, the severity of the phenotype should depend on the fraction that is represented by fully active wt/wt in the healthy carriers and by the monomeric inactive F119L in the patients. However in addition to this, we found that fraction represented by F119L/R141H, although being active, is unstable. For this reason in the cells of the patients the reduced phosphomannomutase2 activity should be primarily due to a lower protein concentration and the genotype F119L/R141H should be responsive to pharmacological chaperones and/or to drugs that act on proteostasis. As a proof of concept we have analyzed fibroblasts derived from two patients carrying the genotype F119L/R141H, those deriving from a patient carrying the genotype F119L/F119L and those derived from two healthy controls matched by age, and we have found that the amount of the protein revealed by western blot is much less in the cells of the patients. We are aware of the fact that this type of analysis should be carried out on a higher number of cases and of controls and more importantly on fresh derived lymphoblasts. It is known that the activity of PMM2 is variable when fibroblasts are analyzed and tend to increase upon passages [7].

To support our finding that the inactive mutant R141H ameliorates the activity of the hypomorphic mutant F119L, we analyzed the literature. We found in the paper by Pirard *et al* [9] that PMM2 activity measured in fibroblasts derived by 19 patients F119L/R141H and 1 patient F119L/F119L are similar although a lower activity was expected for the heterozygous patients.

In conclusion our paper demonstrates that the activity of F119L/R141H is higher than expected due to the promoting protein dimerization by the inactive mutant. The mutual assistance between the mutants is well described borrowing the epigram about the blind man and the lame from Plato the Younger: " A blind man carried a lame man on his back lending him his feet and borrowing from him his eyes". This effect might not be restricted to PMM2, but might occur with other proteins that are active as dimers.

The reduction of total activity found in the patients carrying F119L/R141H can be attributed to instability and a lower amount of the protein in the cells, better than to an intrinsic reduction of the specific activity. In turn, this opens the possibility that these patients, who represent a large share of the total in Northern Europe, can benefit from a therapy with pharmacological chaperones or drugs that affect protein proteostasis.

## Abbreviations

PMM2-CDG: Congenital disorder of glycosylation due to mutations of phosphomannomutase2
PMM2: phosphomannomutase2
Glc-1,6-P_2_: glucose1,6-bisphosphate
Glc-1-P: glucose 1-phosphate
EC50: Median Effective Concentration
RP-HPLC-ESI-MS: reverse phase high performance liquid chromatography electrospray ionization mass spectrometry
wt: wild type
FRET: Förster/Fluorescence resonance energy transfer
